# Exploring the Role of English as a Foreign Language Receptive Skills and Learning Strategy Usage in the Ability to Acquire and Apply Knowledge at the Beginning of Higher Education

**DOI:** 10.3389/fpsyg.2022.808546

**Published:** 2022-05-30

**Authors:** Andrea Magyar, Anita Habók, Gyöngyvér Molnár

**Affiliations:** ^1^MTA–SZTE Digital Learning Technologies Research Group, Szeged, Hungary; ^2^Institute of Education, University of Szeged, Szeged, Hungary

**Keywords:** EFL receptive skills, learning strategy usage, knowledge acquisition, knowledge application, higher education

## Abstract

Foreign language learning plays a prominent role in the world today not only for communication across borders, but also for the potential benefits of other learning skills. The main objective of this research is to examine and explore the relationship between first-year full-time undergraduate students’ (*N* = 1,257) English as a foreign language (EFL) reading and listening achievement and learning strategy preferences in relation to knowledge acquisition and knowledge application. Our results show that students achieved significantly better on listening tasks than on reading tasks and that their knowledge acquisition performance was higher than their knowledge application achievement. The majority of the participants reported that they usually or always employ learning strategies, with the most preferred strategy type being the control strategy. The structural model shows that language learning, and knowledge acquisition and application are strongly interrelated; moreover, the level of use of memorization and elaboration strategies directly affects both knowledge acquisition and application skills. This suggests that EFL learning significantly influences the development of knowledge acquisition and knowledge application, which are essential in a range of areas in education and society today.

## Introduction

Foreign language proficiency is a requirement for students in many countries today. In Hungary, even secondary school students are required to pass at least one B2-level foreign language exam, and a language certificate is also a prerequisite for receiving a degree. Most universities offer courses taught in a foreign language, which also require an adequate level of language skills. However, as research has shown, language learning has potential benefits not only for language studies, but also for a number of learning and study skills by affecting numerous brain functions and connecting the left and the right hemispheres, thus enabling the transmission and integration of information between them (Grosjean and Li, 2013; Li, 2015). Foreign language skills can thus facilitate knowledge acquisition and knowledge application, which are indicators of problem-solving skills (Wüstenberg et al., 2014), in a wide range of subjects and at various levels.

Effective knowledge acquisition also depends on a number of cognitive and affective factors, techniques and methods. One of the most researched areas is the effect of learning approaches and techniques, in which learning strategies play a dominant role. Students who adopt and master effective learning strategies become more efficient and successful learners not only of language, but in other areas as well (Oxford, 2017; Rovers et al., 2018; Habók and Magyar, 2018a, 2019, 2020). Language learning strategies have recently been in the focus of language studies (Zhang et al., 2019), with the most often used taxonomy for these strategies having been developed by Oxford (1990). However, there are studies that have created other taxonomies (Habók and Magyar, 2018b; Zhang et al., 2019). The OECD PISA survey also aimed to identify strategies that can help learners to become more aware and effective readers (OECD, 2010). It defined learning strategies as approaches to learning and identified five main components: (1) understanding and remembering, (2) summarizing, (3) memorization, (4) control and (5) elaboration strategies.

To gain a more in-depth understanding of the links between language learning and the factors noted above, it is extremely important to discover how and to what extent these factors are linked to each other and to language learning skills. A relatively large number of studies have focused on these factors of language learning; however, only a few have investigated the direct relations between them (Nikolov and Csapó, 2018; Taheri et al., 2019; Lin et al., 2021; Malpartida, 2021), and even fewer of them have examined their effect on each other. In our study, we explore how certain factors are related. We investigate university students’ English as foreign language (EFL) skills in relation to their learning strategies and their ability to acquire and apply knowledge in uncertain situations. Our aim is to map the relations between these factors, specifically, how EFL reading and listening skills are related to the elaboration, memorization and control learning strategies and the effect they have on their knowledge acquisition and knowledge application skills.

### Theoretical Background

#### EFL Skills: Reading and Listening

In recent years, the study of second language acquisition has been dominated by cognitive psychological perspectives. Foreign language proficiency assumes a large number of underlying skills that enable learners to communicate properly. Complex skills consist of a number of lower-level component skills. Traditionally, the four basic skill categories are listening and reading (the receptive skills) and speaking and writing (the productive skills). However, it is evident that each of these encompasses a large number of sub-skills (Schmitt, 2010).

The reason why the receptive skills were employed in this study is that it is much more reliable to measure them in a digital environment than it is to measure productive skills. Closed-ended item types are preferred on automatized computerized tests. Also, productive skills stem from receptive skills (*cf*. input hypothesis of Krashen, 1985). Together, they can be regarded as basic component skills. Research has also demonstrated that the four basic skills are highly interdependent; the results can therefore be generalized for all the basic skills (Krashen, 1989; Kent and Wanzek, 2016; Pae and O’Brien, 2018; Yuzar and Rejeki, 2020). According to Yuzar and Rejeki (2020), listening is the most prominent skill, one which greatly influences other language proficiency skills.

Reading and listening are regarded as basic receptive skills, as they provide sources of input for language learners. During the language learning process, students first tend to acquire receptive skills and then develop productive skills. Later, the two encompass each other and play a fundamental role in enhancing other skills. The most important difference between reading and listening is that listening provides less support for the learner than written texts. Listening is a real-time process; there is no way to hear the information again many times. However, this does not mean that the reader or the listener is only a passive interactant, as both skills require active participation from the learners (Schmitt, 2010).

Goodman (1967) interpreted reading as a “psycholinguistic guessing game” (p. 2), in which the reader attempts to decode, extract and construct meaning from his or her own perspective, while actively interacting with the text (Goodman, 1967; Sheth, 2015; Wong, 2020). The final message depends on both the writer’s intentions and the reader’s interpretation. Reading comprehension is thus an interactive process between the reader and the text, and, the reader engages in varying levels of language skills, techniques and strategies during this interaction (Yapp et al., 2021).

Listening is also regarded as an active process, since it involves active participation among listeners to comprehend and construct the meaning of what is being said. It also requires a number of skills to understand what others are saying. These involve recognition of sound articulated by the speaker, perception of intonation patterns, construal of the essence of what is being said, understanding the vocabulary and grammar patterns, and placing the information into context (Kothacheruvu, 2014; Yuzar and Rejeki, 2020; Dong et al., 2021).

A number of studies have investigated the relationship between reading and listening and have reported a strong link between them (Graham, 2006; Babayiğit, 2014; Yuzar and Rejeki, 2020; Ha, 2021). Yuzar and Rejeki (2020) found that listening was the dominant skill and that it has a strong correlation with all other language proficiency skills. They also claimed that listening was the most difficult skill to acquire due to its complexity in the language learning process. Ha (2021) noted that a very small number of studies have analysed the multiple connections between specific types of vocabulary knowledge, such as phonological and orthographic knowledge, and listening and reading comprehension, and he confirmed a significant correlation between reading and listening. Babayiğit (2014) also emphasized that listening is strongly tied to foreign language reading comprehension. Graham (2006) examined the mutual relationships between the two receptive skills and pointed out that the reading and listening modes are not symmetrical, with students completing a reading comprehension task more successfully than a listening comprehension task with the same text.

Some studies have investigated both the interrelatedness and the effects of reading and listening skills (Wolf et al., 2019; Miao, 2021; Jeakel et al., 2022). Wolf et al. (2019) found a strong correlation and mutual predictive power for the two receptive skills. Jeakel et al. (2022) also investigated the effect of EFL on language proficiency in the areas of reading and listening. They concluded that there is a significant relationship between variables based on reading and listening comprehension subtasks. Miao (2021) integrated the two skills and found a positive effect not only on students’ EFL performance, but also on their strategy use.

#### Learning Strategies

Language learners need to employ certain techniques, methods or strategies to develop their foreign language skills. Language learning strategies are “complex, dynamic thoughts and actions, selected and used by learners with some degree of consciousness in specific contexts in order to regulate multiple aspects of themselves … for the purpose of (a) accomplishing language tasks; (b) improving language performance or use; and/or (c) enhancing long-term proficiency” (Oxford, 2017, p. 48). Oxford (1990) developed the most comprehensive taxonomy of language learning strategies, involving three different strategy types, cognitive, affective and sociocultural-interactive, with each subsumed under their respective metastrategies, metacognitive, meta-affective and meta-sociocultural-interactive strategies (Oxford, 2011, 2017). As she dealt with language learning strategies in general, her model did not elaborate on specific language skills. O’Malley and Chamot (1990) developed a model specifically for foreign language reading strategies, in which they classified reading strategies into three main groups, cognitive, metacognitive and socio-affective strategies, depending on the level or category of the thinking processing involved. Cognitive reading strategies operate in direct interaction with the written text and facilitate comprehension. They include underlining, guessing from context and summarizing. Metacognitive strategies involve monitoring, planning and evaluating, which can occur in any of the phases of the reading process. Socio-affective strategies are related to social mediation activities and transactions with others, such as comprehension checks and requests for clarification.

Mokhtari and Reichard (2002) placed reading strategies in three domains: (1) global, (2) problem-solving and (3) support strategies. Global strategies refer to techniques that are used to monitor reading. Such strategies comprise predicting, previewing, scanning and skimming the text. Problem-solving strategies facilitate the reading of complicated texts. These include guessing, visualizing and reading at a slow pace. Support reading strategies (SUP) are employed to aid readers in enhancing their text comprehension. For instance, note-taking during reading, summarising and paraphrasing the text to facilitate comprehension.

Other researchers have built their taxonomies based on the reading process involved, that is, reading strategies employed before, during and after the reading process. Before reading, effective foreign language readers use their prior knowledge of the topic, they predict the probable meaning of the text, or they skim or scan the text. While they read, they continuously monitor their understanding and link the information they are reading to their prior experience and knowledge. After reading, they reflect on their ideas and extend their understanding of the text (Baker and Boonkit, 2004; Habók and Magyar, 2019; Oo et al., 2021). Habók and Magyar (2019) found moderate correlations between reading strategy use and reading test results. Reading strategies were directly influenced by English language attitude. The indirect effect of general English proficiency was explained through English language attitude and reading strategy use.

Like reading strategies, there are various categorizations of listening strategies. Bao and Guan (2018) developed a classification of listening strategies of three main types: cognitive, metacognitive and socio-affective strategies. Cognitive strategies are the most essential for listening comprehension as they support students in monitoring and controlling their mental processes, thus enhancing mental abilities and processes tied to knowledge, such as using linguistic features to comprehend the text. They include predicting, inferring, interpreting, storing and recalling information. Metacognitive strategies aid in thinking about one’s mental processes and learning methods. They are central to managing and monitoring students’ strategy use and help to plan, monitor and interpret certain mental processes while listening. Socio-affective listening strategies foster interaction with peers and facilitate listeners in eliminating negative feelings, for instance, anxiety (Bao and Guan, 2018). Strategy research has pointed out the necessity of teaching listening strategies for the development of L2 listening proficiency (Graham, 2006; Graham and Macaro, 2008). Wakamoto and Rose (2021) developed a three-factor model involving the cognitive, metacognitive and practice strategy fields. They also established the predictive role of listening strategies on listening comprehension among university students.

OECD (2010) also developed a framework for measuring certain learning strategies. The framework involved elaboration, memorization and control strategies. Elaboration strategies indicate how deeply learners relate new material to what they already know, how deeply they can tie the new material to materials studied in other subjects, and how they determine whether a piece of information is useful in the real world or not.

The OECD (2010) results underlined that elaboration strategies and reading performance are strongly related. The difference in performance among students who use mostly elaboration strategies and that of those who use them at a low level varied across countries. This may be due to the different teaching and learning methods used in the various countries.

Memorization strategies aid in storing new information in memory without further processing. Research (OECD, 2010) suggests that memorization strategies do not produce in-depth understanding, as they do not help develop students’ skills to deduce the underlying meaning and message of stored information in order to integrate new knowledge and prior assumptions. Results also indicate that memorization strategies relate positively to reading performance in some European countries, while use of memorization strategies is associated with lower reading performance in other countries. The report also pointed out that students who used memorization strategies more frequently and those who used those strategies to the same extent as the OECD average performed equally well in reading. In certain countries, students reported a greater use of memorization strategies, yet they were poorer readers than those whose use was closer to the OECD average.

Control strategies assist in assuring that learners reach their learning goals. They also help to decide what the students have already learned and pay attention to what they still need to learn or what they do not understand. According to the PISA results, an average of 5–10% of the variation in students’ reading performance can be explained by the use of control strategies (OECD, 2010). The PISA results also indicated that control strategies are essential for effective self-regulation of learning, as they help students adapt their learning to the particular task at hand. To sum up, PISA showed that students’ learning strategy use could be associated with reading performance.

#### Knowledge Acquisition and Knowledge Application Skills

Language learners often face complex, meaning-focused tasks that involve a cumulative sequence of cognitive processes, situation-specific thinking, and activating and reconstructing links between recent and new knowledge stored in memory. These complex tasks require higher-order skills that are not available at the beginning of the process and rather need to be constructed during it (Molnár et al., 2021; Nicolay et al., 2021). Knowledge acquisition and knowledge application also entail the mobilization of certain motivational and creative skills to find a solution to the unfamiliar task (Lesh and Zawojewski, 2007).

Both knowledge acquisition and knowledge application cover high-level cognitive processes that involve effective coordination of certain other elementary cognitive operations, such as attention, memory use, perception and learning (Greiff et al., 2012; Zhang and Zhang, 2020). Knowledge application includes much more than a simple reproduction of knowledge acquired. It also entails the utilization of certain practical and cognitive skills, creativity and other motivational and affective factors, for instance, attitudes, beliefs, motivation and values (OECD, 2013; Dörner and Funke, 2017).

Some studies have examined how knowledge acquisition and knowledge application skills are related to certain language skills. Research has stressed that students who learn a second language show better problem-solving skills and higher-order thinking skills than those who do not learn foreign languages (Marcos, 2001). Nikolov and Csapó (2018) examined the link between inductive reasoning skills and achievement on English reading comprehension tests and found a strong correlation between them. Csapó and Nikolov (2009) also pointed out that a cognitive contribution to foreign language proficiency is a significant factor but that this strong connection consistently weakens over the years.

Although an extensive body of literature has emerged to examine the benefits of foreign language learning and its relations to other factors, only a portion of it has investigated how foreign language learning promotes the development of other cognitive skills. A number of questions have therefore remained unanswered. First, the interrelationship between the factors noted above and their role in the foreign language learning process has not yet been ascertained. Second, there is a lack of empirical studies on how certain language skills influence the development of certain problem-solving skills in the knowledge acquisition and knowledge application processes. Third, the exact role of strategy use in this process is also unknown. We are seeking answers for these gaps.

### Research Questions

In this research, we studied first-year full-time students’ EFL reading and listening skills, knowledge acquisition and knowledge application as a measure of problem-solving skills and strategy use. In addition, we examined the path of their relations as components of successful EFL learning.

We addressed the following research questions:

(1) What is the developmental level of first-year full-time students’ EFL reading and listening achievement, knowledge acquisition and application, and what types of learning strategies do they prefer?
(2) What are the relationships between first-year full-time students’ EFL reading, listening and knowledge acquisition and knowledge application and learning strategies?
(3) How do students’ EFL reading and listening skills influence their knowledge acquisition, knowledge application and learning strategy use?

## Materials and Methods

### Participants

The overall sample was formed of students from 11 university faculties located at a university in southern Hungary. 1,257 students participated in the measurement in total, with 42.3% of them being male (*N* = 532). Designed for full-time students, the study is part of a large-scale institutional longitudinal project. First-year students were invited to participate in the institutional measurement, which is conducted at the very beginning of their first term. Students’ participation was voluntary, and they received one credit for participating. First, students were informed of the main objective of the research and notified that they would complete tests and questionnaires. After the data collection, they received very detailed personalized feedback (a 15-page PDF document per person) with information on their achievement with faculty and university levels as reference points.

### Instruments

The study aims to identify first-year university students’ knowledge and skills in EFL, knowledge acquisition and knowledge application as a measure of problem-solving skills and learning strategies. When constructing the tasks, we made sure that they had different difficulty levels. As regards listening tasks, students heard two authentic texts twice *via* earphones. While listening to the text, they completed multiple-choice tasks and chose the correct answer from three options. On the reading tasks, students read two texts and filled in gaps with nouns, verbs, adverbs or adjectives. The text consisted of B1- and B2-level listening and reading tasks. The reliability analysis of the reading and listening test sessions showed that all fields have good and high reliability.

Knowledge acquisition and knowledge application were measured within the confines of complex problem-solving using the MicroDYN approach. The measurement of these kinds of complex problems is typically achieved by computer-simulated systems, or problem scenarios, which contain several interrelated variables. The problem-solving process consists of two phases: a knowledge acquisition phase and a knowledge application phase (Molnár et al., 2013; Greiff et al., 2021). In the first phase, problem-solvers need to comprehend the working method of the system and identify how the variables are interconnected. In this exploration phase, they can change the values of the variables and detect the changes in the system, i.e., in the output variables, to discover the rules and relations between them. Meanwhile, they employ a number of combinational and classification operations and skills. At the very end of the first phase of the problem-solving process, they define and visualize the relations they have detected on a concept map provided in the simulated problem scenario. In the second phase, the knowledge application phase, they apply their newly acquired knowledge and bring the system to a given state; that is, they reach the target values of the output variables by manipulating the input variables. The right concept map is provided within the problem scenario to avoid item dependence (Greiff and Funke, 2009; Greiff et al., 2012). Based on empirical evidence (see, e.g., Greiff et al., 2013; Molnár et al., 2017, 2021), complex and interactive scenarios are good measures of knowledge acquisition and knowledge application.

The test contains 20 items with different difficulty levels. Students needed no factual knowledge from school to be able to deal with the problems. These problems did not measure how students apply rules that might be involved in a school assignment, but how they engage in exploration and learning in a new situation and how effectively they can apply the knowledge they have acquired. Both types of items that measure knowledge acquisition and knowledge application proved to be reliable in the university sample (Table 1).

**Table 1:** Internal consistency (CRB) of reading, listening, knowledge acquisition and knowledge application items.

Cognitive tasks	CRB *α*
Listening tasks (20 items)	0.879
Reading tasks (20 items)	0.962
Knowledge acquisition (10 items)	0.836
Knowledge application (10 items)	0.832

We also used a questionnaire, which was originally designed for the PISA studies (Artelt et al., 2003; OECD, 2013). As for learning strategies, students rated statements on elaboration, memorization and control strategies on a five-point Likert scale, where the answers ranged from ‘never’ to ‘always’. In the field of elaboration strategies, students marked whether they link new information to prior knowledge and how they ascertain whether the information could be useful in the real world. For example, ‘When I study, I try to understand the material better by relating it to my own experience’. Statements about memorization strategies determined how frequently students apply memorization and recitation of learning materials. For instance, ‘When I study, I read the text so many times that I can recite it’. Finally, statements in the control strategies category investigated whether students attempt to ascertain what they already know, have learned or did not understand or whether they look for new information. For example, ‘When I study, I make sure that I remember the most important points in the text’. We concluded that the measurement tools worked reliably (Table 2).

**Table 2:** Internal consistency reliability (CRB) for strategy fields.

Strategy fields	CRB *α*
Elaboration	0.601
Memorization	0.645
Control	0.689

### Design and Procedure

After the students were informed of the objectives of the research, they had the opportunity to register and select a date for the measurement. Students completed the tests and questionnaires in the eDia online system (Csapó and Molnár, 2019). The venue for data collection was the university information centre, where it was possible to test 150 students at the same time. Trained measurement supervisors conducted the measurement sessions and answered students’ questions. Sixty minutes were provided for each measurement tool. Students were able to complete the two measurement tools in the same session, that is, 2 × 60 min. They also received immediate feedback on their results at the end of the measurement. Two weeks after the data collection, students received detailed personalized feedback on their knowledge and skills, highlighting the areas in which they need to develop and in which they performed excellently. Moreover, it was also indicated how they achieved compared to their peers. To sum up, a figure for each of the measured fields was prepared for each student, in which their lowest and highest achievement was indicated along with the average performance of the sample.

### Data Analyses

First, we used IBM SPSS statistics 22.0 for classical test analysis to examine internal consistency reliability, mean, standard deviation, frequencies and correlation. Second, structural equation modelling (SEM) was conducted to analyse the relations between EFL reading and listening skills, knowledge acquisition and knowledge application as a measure of problem-solving, and learning strategy use. The IMB AMOS 24.0 software package was used to evaluate our model, and the Chi-square test, comparative fit index (CFI), Tucker–Lewis Index (TLI), root mean square error of approximation (RMSEA) and normed fit index (NFI) were employed to analyse fit indices. CFI, TLI and NFI can range between 0 and 1, with a cut-off value of 0.90 indicating an acceptable model fit. The RMSEA value also ranges between 0 and 1, with lower values representing a higher model fit. A value of 0.06 or less is generally indicative of a good model fit; however, a value of 0.08 or less is still an acceptable model fit (Kline, 2015).

## Results

### Descriptive Analysis

**Figure 1:**
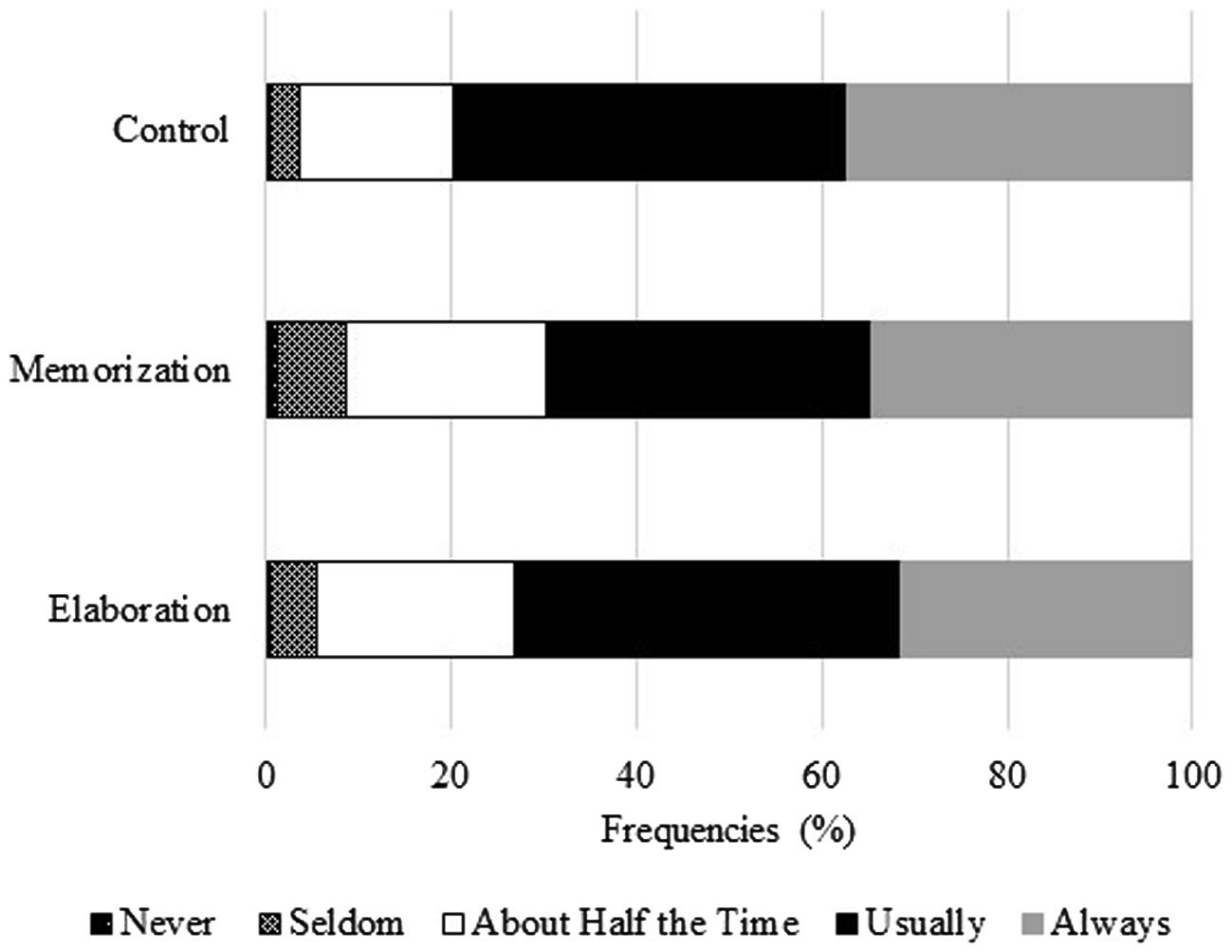
Frequencies of students’ responses.

Means were calculated for each task type. Means and standard deviations are presented in Table 3. The mean scores for the cognitive tasks vary between 73 and 36%p. This indicates that students had difficulty with reading tasks and knowledge application. As regards strategy use, students used control strategies the most; however, strategy use shows similar trends. Students gave the highest rating to statements that dealt with recalling prior knowledge and tying it to new information they had learned (*M* = 4.27). They also gave a high rating to checking whether they learned the most important things (*M* = 4.23). Students rated memorization strategies the lowest, meaning they do not memorize all the learning material to recall it. They likely focus on the details they feel they need to learn (Figure 1).

**Table 3:** Means for strategy fields.

Fields	*M* (%p)	SD
*Cognitive tasks*		
Listening task	73	18
Reading tasks	55	29
Knowledge acquisition	55	26
Knowledge application	36	25
*Strategies*		
Elaboration	69	17
Memorization	69	19
Control	75	16

We analysed frequencies to provide a more detailed overview of students’ strategy use. On the whole, it can be concluded that the majority of our sample ‘usually’ or ‘always’ apply learning strategies. More than 50% of the sample reported frequent strategy use.

### Multivariate Analysis

We analysed the correlation between reading and listening knowledge, knowledge acquisition and knowledge application, and related strategies. We found significant correlation coefficients in the majority of cases (*r* = 0.75 to −0.26). The strongest relations were found between the reading and listening variables. As regards strategy use, the greatest correlation was found between the elaboration and control strategies (*r* = 0.42). The memorization strategies only showed significant positive relations with the control (*r* = 0.38) and elaboration (*r* = 0.17) strategies. The correlation coefficients for the memorization strategies were negative but significant (*r* = −0.26 to −0.11) with the other factors. The correlation was moderate between the receptive skills and knowledge acquisition and application skills (*r* = 0.27–0.13; Table 4).

**Table 4:** Correlations between language skills, levels of knowledge acquisition, and knowledge application and learning strategies.

	1	2	3	4	5	6	7
1. Listening task	1						
2. Reading tasks	0.75	1					
3. Knowledge acquisition	0.27	0.25	1				
4. Knowledge application	0.13	0.13	0.43	1			
5. Elaboration	0.08	0.12	0.09	0.08	1		
6. Memorization	−0.11	−0.11	−0.26	−0.15	0.17	1	
7. Control	n.s.	n.s.	n.s.	n.s.	0.42	0.38	1

*Correlation is significant at the 0.01 level*.

We developed a structural model to synthesize our results and consider the factors under investigation with significant correlations. Two exogenous constructs (reading and listening skills) and five endogenous constructs (the factors of learning strategies and knowledge acquisition and knowledge application) were employed in this model. Nine structural relations were discovered between the constructs: the direct effect of reading skills on memorization and elaboration strategies, the effect of listening skills on knowledge acquisition and knowledge application, the effect of memorization and elaboration strategies on both knowledge acquisition and knowledge application, and the effect of control strategies on elaboration strategies.

**Figure 2:**
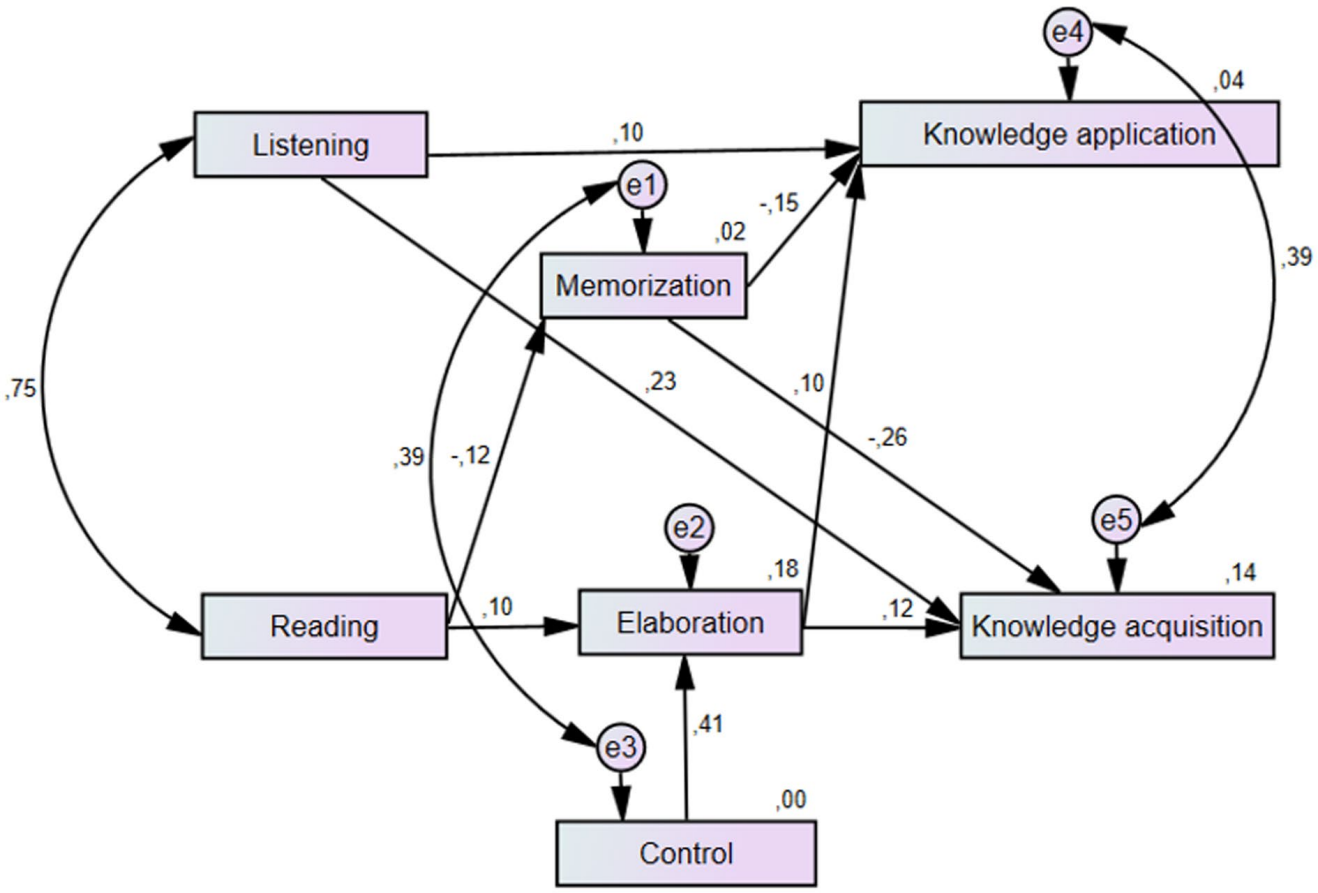
Structural model for English as a foreign language (EFL) receptive skills, learning strategy use, and knowledge acquisition and application.

The structural equation model shows the predictive powers of reading and listening skills on knowledge acquisition, knowledge application and related learning strategies. The fit indices of the SEM met good levels (Chi-square = 9.541; *d* = 9; *p* = 0.389; CFI = 1.000; TLI = 0.999; NFI = 0.995; RMSEA = 0.007). Figure 2 indicates the standardized estimates on regression paths in the model. Positive coefficients show positive directions, and negative coefficients starting from knowledge acquisition indicate a negative effect on the memorization strategy. All paths are significant at *p* < 0.05.

The structural equation model demonstrated that different types of skills have different predictive power on each other. Specifically, reading knowledge predicts memorization (*β* = −0.12) and elaboration (*β* = 0.03). Listening knowledge is significantly predictive of knowledge acquisition (*β* = 0.23) and knowledge application (*β* = 0.10). Memorization predicts knowledge application (*β* = −0.15) and knowledge acquisition (*β* = −0.26). Elaboration has a similar effect on knowledge application (*β* = 0.10) and knowledge acquisition (*β* = 0.12), while control strategies have a considerable effect on control strategies (*β* = 0.41). The double-headed arrows indicate strong correlations between listening and reading, knowledge acquisition and application, and memorization and elaboration strategies (Figure 2).

## Discussion

Our study investigated first-year full-time undergraduate students’ EFL receptive skills, their strategy use, knowledge acquisition and knowledge application, and their relations with and effects on each other. Our first research question focused on how students performed on EFL reading, listening, knowledge acquisition and knowledge application, and how frequently they employ certain learning strategies. Our findings show that students performed significantly better on listening tasks than on reading tasks, which is in line with results of Yuzar and Rejeki (2020). The students’ knowledge acquisition performance was far better than their knowledge application performance, which is consistent with Dörner and Funke (2017). Meanwhile, it can be concluded that the majority of the sample usually or always apply learning strategies. More than half of the sample reported that they frequently use the strategies measured. It is very useful that students employ all three kinds of strategies. However, frequent strategy use in memorization is problematic, as memorization in this context refers to rote learning and not to understood knowledge, which was also reinforced by Rovers et al. (2018). That the majority of students apply elaboration and control strategies is a welcome finding. These results are consistent with those of other studies that have also observed frequent use of elaboration and control strategy use (Biggs and Tang, 2011; OECD, 2013; Laird et al., 2014).

Our research also discovered significant relations between the students’ EFL receptive skills, level of knowledge acquisition and knowledge application, and learning strategy use. We found strong links between reading and listening skills. This reinforces the strong interrelatedness of these skills, which has been confirmed in many other studies (Kent and Wanzek, 2016; Pae and O’Brien, 2018; Yuzar and Rejeki, 2020). Research of Yuzar and Rejeki (2020) also discovered significant correlations between reading and listening and pointed out the prominence of this skill, as it can greatly influence other language proficiency skills. Knowledge acquisition and application also showed a significant relationship, which has likewise been found in PISA measurements (OECD, 2013). Learning strategy factors also correlated highly with each other similarly to study of Habók and Magyar (2018a). The strongest relation was demonstrated between the elaboration and control strategies, which is consistent with the PISA results (Artelt et al., 2003).

We also analysed the relations between learning strategies, knowledge acquisition and knowledge application, and found significant but moderate relationships between them. Both knowledge acquisition and knowledge application skills involve the use of such subskills as combinational and classification operations and skills that greatly influence student performance on these kinds of tasks. We have observed trends similar to those identified by Csapó and Molnár (2017), who also found significant relations between problem-solving strategies as a measure of knowledge acquisition and particular strategy use. The relationship between knowledge acquisition and application with elaboration and control strategies is positive, with memory strategies being significantly negative. They reinforce the fact that students who use their exploratory skills are better in acquiring and applying their knowledge as well. The constant monitoring function is common to both processes. However, rote learners who memorize a great deal probably do not look for meanings in the learning task and do not employ their reasoning skills; therefore, less stress is placed on a meaningful learning process (Habók, 2012; Rovers et al., 2018; Habók et al., 2019).

We developed a structural model to explore the directions between the factors under examination. We used it to reliably synthesize our results and show the direct and indirect effects between the factors. The model enabled us to identify more significant impacts of EFL language skills on knowledge acquisition and knowledge application, while positive effects were also observed on the different kinds of learning strategies. Among the learning strategy components, the greatest effects were those of the control strategies that monitor the learning process and learning output on the elaboration strategies. The effect values within the model are all significant but moderate. Both memorization and elaboration directly affect both knowledge acquisition and application. This implies that EFL learning significantly influences the development of knowledge acquisition and knowledge application, which are essential in education and society today (OECD, 2013; Csapó and Funke, 2017; Csapó and Molnár, 2017; Habók and Magyar, 2018a).

## Conclusion and Pedagogical Implications

Our research used descriptive statistics and SEM to examine first-year undergraduate students’ EFL reading and listening skills, knowledge acquisition, knowledge application and learning strategy application. The main outcome of our research is that it has confirmed findings from previous studies and identified some new relationships between the constructs under investigation as well.

Our results showed that language learning, knowledge acquisition and knowledge application are closely related. It is therefore very important to consciously train students to improve their skills in educational settings. As EFL receptive skills are highly correlated with other language skills, such language learning is beneficial not only for communication, but also for the development of other skills and competencies. Twenty-first-century skills, such as knowledge acquisition and knowledge application, form the basis for many other areas of knowledge application and knowledge application processes. Our findings also confirm the significance of teaching learning strategies, as they directly influence knowledge acquisition and knowledge application as a measure of problem-solving skills. The findings also highlight the fact that rote memorization negatively influences both knowledge acquisition and application skills.

The main pedagogical advantage of our research is that it highlights the significance of learning strategies in the field of language learning in classroom settings. It also illuminates the importance of knowledge acquisition and application in the language learning process. Teachers can draw conclusions on how and why students should apply certain strategies in their foreign language listening or reading exercises and how to transfer them to their other studies.

The results of our research pave the way for subsequent investigations not only in higher education, but also at lower levels of education. The findings may be applicable both in secondary and primary schools. Furthermore, not only the receptive skills, but also the productive skills, that is, writing and speaking, are recommended as a focus of investigation in future research. Other areas may be involved, such as mathematical reasoning skills and the role of certain affective factors. Affective factors can include attitude towards language learning or motivational issues.

### Limitations

We also note certain limitations in our research. First, we only involved first-year undergraduate students from one institution, so our results cannot be generalized. Second, the reading and listening tasks were built on multiple-choice answers and consisted of the same structure in both areas, thus making it easy to evaluate the tasks immediately and provide feedback to the students. However, there may be students who are more successful with other types of tasks. For example, some may perform more successfully on essay tasks. Third, the learning strategy questionnaire did not contain enough statements to employ a sophisticated measurement of these constructs. Fourth, students might have thought that their answers would have an impact on their university studies, so they might have rated their strategy use higher—even though their responses were for research purposes only.

## Data Availability Statement

The original contributions presented in the study are included in the article/supplementary material, and further inquiries can be directed to the corresponding author.

## Ethics Statement

Ethical review and approval was not required for the study on human participants in accordance with the local legislation and institutional requirements. Written informed consent for participation was not required for this study in accordance with the national legislation and the institutional requirements.

## Author Contributions

GM designed the measurement tools and collected data. AM and AH managed the data processing procedure. All authors wrote the manuscript and made intellectual contributions to the writing. All authors approved the final version of the manuscript for publishing.

## Funding

This research was supported by a Hungarian National Research, Development and Innovation Fund grant (under the OTKA K135727 funding scheme) and a Research Programme for Public Education Development of the Hungarian Academy of Sciences grant (KOZOKT2021-16).

## Conflict of Interest

The authors declare that the research was conducted in the absence of any commercial or financial relationships that could be construed as a potential conflict of interest.

## Publisher’s Note

All claims expressed in this article are solely those of the authors and do not necessarily represent those of their affiliated organizations, or those of the publisher, the editors and the reviewers. Any product that may be evaluated in this article, or claim that may be made by its manufacturer, is not guaranteed or endorsed by the publisher.
